# Understanding India, globalisation and health care systems: a mapping of research in the social sciences

**DOI:** 10.1186/1744-8603-8-32

**Published:** 2012-09-10

**Authors:** Ramila Bisht, Emma Pitchforth, Susan F Murray

**Affiliations:** 1 Centre of Social Medicine and Community Health, School of Social Sciences, Jawaharlal Nehru University, New Delhi 110067, India; 2 RAND Europe, Westbrook Centre, Milton Road, Cambridge, CB4 1YG, UK; 3 King's College London, James Clerk Maxwell Building, Waterloo Road, London, SE1 8WA, UK

**Keywords:** Social science research, Globalisation and healthcare, India, State of the art literature review, Transnational, Trade

## Abstract

National and transnational health care systems are rapidly evolving with current processes of globalisation. What is the contribution of the social sciences to an understanding of this field? A structured scoping exercise was conducted to identify relevant literature using the lens of India – a ‘rising power’ with a rapidly expanding healthcare economy. A five step search and analysis method was employed in order to capture as wide a range of material as possible. Documents published in English that met criteria for a social science contribution were included for review. Via electronic bibliographic databases, websites and hand searches conducted in India, 113 relevant articles, books and reports were identified. These were classified according to topic area, publication date, disciplinary perspective, genre, and theoretical and methodological approaches. Topic areas were identified initially through an inductive approach, then rationalised into seven broad themes. Transnational consumption of health services; the transnational healthcare workforce; the production, consumption and trade in specific health-related commodities, and transnational diffusion of ideas and knowledge have all received attention from social scientists in work related to India. Other themes with smaller volumes of work include new global health governance issues and structures; transnational delivery of health services and the transnational movement of capital. Thirteen disciplines were found represented in our review, with social policy being a clear leader, followed by economics and management studies. Overall this survey of India-related work suggests a young and expanding literature, although hampered by inadequacies in global comparative data, and by difficulties in accessing commercially sensitive information. The field would benefit from further cross-fertilisation between disciplines and greater application of explanatory theory. Literatures around stem cell research and health related commodities provide some excellent examples of illuminating social science. Future research agendas on health systems issues need to include innovative empirical work that captures the dynamics of transnational processes and that links macro-level change to fine-grained observations of social life.

## Introduction

Current globalisation is regarded as a new phase of world integration with increased density and frequency of international or global social interactions relative to local or national ones. In these new dynamics, nation states are influenced by transnational processes occurring on multiple economic, political and cultural levels [1]. Much public health literature still assumes a default model of a ‘healthcare system’ bounded by a nation state which to varying degrees takes responsibility for the four essential functions of service provision, resource generation, financing and stewardship [2], but there is increasing awareness that globalisation processes are shaping local health systems in important ways [3]. In this article we set out to map through the lens of India’s healthcare and its relationship to transnational health care systems, the current extent and form of the social science approaches to an understanding of this field.

India is an illuminating case for which one would expect to find a considerable body of literature. The country has emerged as one of the so-called ‘Rising Powers’ which represent drivers of global economic and social change. Healthcare is one of India’s largest sectors in terms of revenue and employment, and is expanding rapidly. The private sector accounts for more than 80% of total healthcare spending within India [4]. Since ‘liberalisation’ of economic policies in the late 1980s, India has also become increasingly attractive to foreign investors with its low costs and large, English-speaking, workforce. During the 1990s, Indian healthcare grew at a compound annual rate of 16% and today the total value of the sector is more than $34 billion and projected to grow to nearly $40 billion by 2012. The consultancy group Pricewaterhouse Coopers’ *Emerging Market Report on Healthcare in India*[5] listed a number of ‘flourishing market opportunities’ for foreign as well as national companies: medical tourism (often combining elective surgery and aspects of Ayurveda), the emerging health insurance market, telemedicine, healthcare infrastructure expansion (including public-private partnerships and training of healthcare staff), the medical equipment market, and the pharmaceuticals industry (manufacture, research and clinical trials). Indian finance capital and healthcare-related firms are expanding horizons to markets and investment opportunities outside its borders. Indian pharmaceutical production, for example, supplies some 22% of the global generics market, primarily exporting to the US and Europe but also to China, Brazil, Nigeria and Mexico. A significant proportion of the vaccines produced are for developing countries [6].

Despite the notable rapid economic growth India is ranked 134 out of 187 countries in the Human Development Index [7]. India currently has great social and economic inequalities. There are marked disparities among different geographical regions, between social groups, among different income levels and between the sexes. The Indian ‘middle class’ is rapidly growing but over a third of the population live on less than US$1 a day, and around a third of the adult population (34.8%), including over 190 million Indian women remain illiterate [8].

The complex and dynamic relationship between India’s adaption to the new global environment and Indian population heath, health systems and healthcare related industries is one deserving of social science scrutiny. In the 1990s, civil society organisations actively campaigned against the healthcare reforms of the structural adjustment policy and produced considerable writings on the negative impact of this process on health and health services. A few landmark studies at that time charted the patterns of utilisation of health services for acute and long term treatment [9-11] and the costs incurred and its implications for equity [10,12,13]. Initially academic research on these themes mostly reflected critical public health interests, at times influenced by social science perspectives. In recent years this has opened out to include an increasingly diverse range of contributions from across the social science disciplines.

This article reports on a scoping exercise conducted to establish the extent of social science research concerning India, globalisation and healthcare systems. Findings are reported where relevant but we seek primarily to bring together an account of the themes, questions, methods and challenges encountered in social science research, and to identify avenues for future work. It hopes to highlight some of the challenges of a health systems and globalisation agenda and of researching the interface between macro level changes and policies and meso and micro level enactment.

### Literature review process

**Figure 1:**
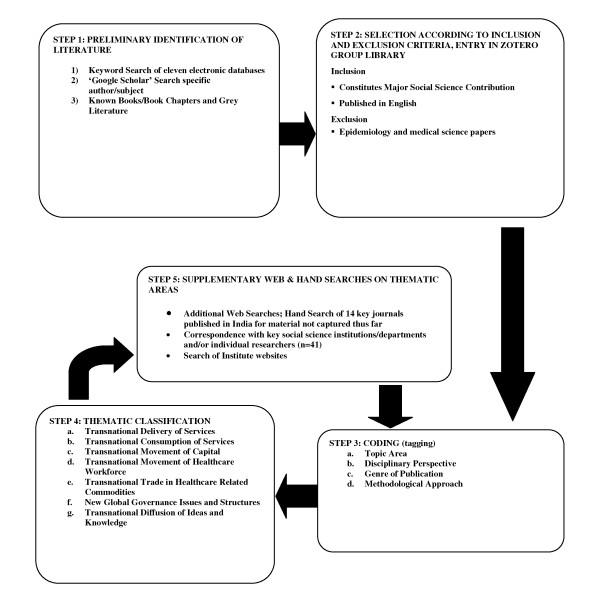
Methodology for Literature Review.

We adapted previous scoping review methodology [14] to devise a five step method of scoping and analysis that could incorporate a broad analytic framework relevant to globalisation and health care (Figure 1). In step 1 conducted in the summer of 2010, eleven electronic bibliographic databases; Scopus, International Bibliography of the Social Sciences (IBSS**);** SocINDEX; Business Source Premier; the PubMed databases (PubMed.gov, PubMed Central and BioMed Central); Applied Social Sciences Index and Abstract (ASSIA); International Political Science Abstracts; Social Sciences Citation Index (Web of Science); Sociological Abstract; Human Resources Abstracts and EconLit were searched. The search terms (see Figure 1) used were derived through a process of refinement. In those databases in which a search combination using the term ‘health’ led to several thousand articles, the modification ‘health care’ was used, in others this proved too limiting and ‘health OR health care’ was employed. 

In step 2, by reviewing the title, journal name and abstracts, we selected articles that: a) constituted a social science contribution to the field and; b) were published in English. We employed a liberal definition of what constitutes a ‘social science contribution’, accepting any of the following - publication in a social science journal; funding received from a social science research council; a first author working in a social science department, faculty or institution; a first author whose discipline was identified as one of the social sciences in the article or whose social science disciplinary background was publically known. The ‘social science’ disciplinary areas were considered to include all those listed as such by the Economic and Social Research Council in the UK (see Table 1). ‘Nursing research’ was included where the research fell into a category of education or human resource management, or had received funding from the ESRC or equivalent funder of social science research. The majority of papers excluded at this stage were epidemiology or medical science papers, many of these relating to the global burden of disease. Research in India is not always published in indexed journals and many scholars publish in books rather than journals. The database searches were supplemented at this point by a search of Google Scholar for specific authors or subjects and relevant books and chapters and grey literature known to the authors were added.

**Table 1:** The social sciences: 19 disciplines listed by the Economic and Social Research Council (ESRC) of the UK


a)	**Area and Development Studies**
b)	Demography
c)	**Economic and Social History**
d)	**Economics**
e)	**Education**
f)	**Human Geography**
g)	Science and Technology Studies
h)	Linguistics
i)	**Management and Business Studies**
j)	Environmental Planning
k)	**Political Science and International Studies**
l)	Psychology
m)	**Social Anthropology**
n)	**Social Policy**
o)	Social Work
p)	**Socio-Legal Studies**
q)	**Sociology**
r)	Statistics
s)	Methods and Computing

The 76 relevant references were entered in Zotero bibliographic software group library. In step 3, initial ‘tagging’ (coding) was undertaken. Articles were classified according to topic area, genre, disciplinary perspective and theoretical and methodological approaches (Figure 1). Topics were initially identified through an inductive approach similar to thematic analysis. These were later rationalised into a number of thematic areas as described below (see Step 4 in Figure 1).

Once the seven thematic areas were identified further web searches and hand searches of bibliographies of key articles were carried out in late 2010 and early 2011 for any material relevant to the themes which had not been captured in the earlier search of the electronic databases (Step 5). Hand searches became a significant element in our methodology as it had become clear that Indian literature is not easily accessible through the electronic databases. A total of 14 journals published from India that were not covered in the electronic databases were hand searched or web-searched. This included *Economic and Political Weekly*, a key journal used by Indian social scientists but not indexed on the above databases. This resulted in 16 additional articles. Finally we wrote to 41 key social science researchers, institutions or departments in India, requesting relevant social science studies and reports on work conducted in the last two decades. Six additional articles were identified in this way, and a search of the websites of these institutions identified 15 further non-duplicate hits. In total 113 articles and chapters were identified as relevant for inclusion in this review.

### Findings

The scoping process resulted in 113 relevant journal articles and chapters. We identified seven thematic areas across this literature. These were: (a) transnational movement of workforce; (b) transnational consumption of services; (c) production, consumption and trade in specific health-related commodities; (d) transnational diffusion of ideas and knowledge; (e) new global governance issues and structures; (f) transnational delivery of services;(g) transnational movement of capital. Table 2 provides an overview of papers in each area in terms of number of articles, publication dates and the social science disciplines represented. Some articles covered more than one theme. The table is effective in showing that certain disciplines, such as economics, are represented across most of the themes whilst sociology and others are concentrated in two or three themes. In total there are contributions from 11 out of the 19 social science disciplines listed by ESRC. The work in each of the thematic areas is summarized below. The summaries aim to describe the main disciplinary and methodological approaches, types of paper and research questions. Particular emphasis is given to highlighting empirical studies or explicit use of social science theory.

**Table 2:** Representation of social science disciplines as listed by the Economic and Social Research Council (ESRC), UK

	**Number of articles**	**Publication date**											
			**Area and Development Studies**	**Economic and Social History**	**Economics**	**Education**	**Human Geography**	**Management and Business Studies**	**Political Science and International Studies**	**Social Anthropology**	**Social Policy**	**Socio-Legal Studies**	**Sociology**
Transnational movement of healthcare workforce	29	1989-2009			✓	✓	✓	✓			✓		✓
Transnational consumption of services	21	2004-2011			✓		✓	✓			✓	✓	
Production, consumption and trade in specific healthcare related commodities	21	1998-2010		✓			✓		✓	✓			✓
Transnational diffusion of ideas and knowledge	19	1998-2010	✓					✓	✓	✓	✓		✓
New global governance issues and structures	14	2000-2010			✓			✓	✓			✓	
Transnational delivery of services	8	2001-2009			✓			✓				✓	
Transnational movement of capital	7	2001-2010			✓						✓		

### Transnational delivery of services

The economist Rupa Chanda has indicated that the healthcare sector is among the most rapidly growing in the world economy, and globalisation of health services is reflected in the growing cross-border delivery and in increasing numbers of joint ventures [15]. Cross border delivery includes shipment of laboratory samples, diagnosis, and clinical consultation via traditional mail channels as well as the rapidly expanding ‘e-health’ services such as electronic delivery of health services such as surveillance, and diagnostics including lab testing, tele-radiology, tele-consultations and remote surgery, and indirect services such as teleconferencing and tele-education, medical transcriptions and claims processing [16].

This area has not as yet received much attention from social scientists. Eight articles making reference to India were identified, all published since 2001, when Chanda’s working paper on ‘Trade in Health Services’ was written for the Commission on Macroeconomics and Health [17]. These comprise three commentary / perspective journal pieces, four concept papers, and one literature review paper, using perspectives variously from Socio-legal studies, Economics and Management and Business Studies. The journals in which they were published ranged from public health to topic specialist, to social science publications. There was a lack of empirical studies in this area.

Smith et al’s overview in the Lancet on trade in health-related services employs the GATS four models of service delivery as a classification for structuring their discussion [16]. In a thoroughly researched article they indicate that overall figures on the volume of such trade are vague, but that existing data indicates that India, along with the Philippines and Cuba, leads in exportation of medical-transcription services, tele-pathology, and tele-diagnostic services. The Apollo Group, the world’s third largest private health provider, is given as an example with activities ranging from provision of regional telemedicine services from centres in South and Eastern Asia, to provision of back office services to US organisations for billing, documentation of clinical and administrative records, coding of medical processes and insurance claims processing. The authors lay out a considerable research agenda in concluding that the implementation and use of e-health raises issues about the recognition of credentials and licensure; legal liability and malpractice considerations, including provider insurance coverage; provider remuneration; patient privacy and confidentiality; the existence of enabling infrastructure and compatibility of standards in areas such as data, images, and medical records; infrastructure and future operating costs; and the quality and appropriateness and fragmentation of care.

Several of the articles identified dealt with the specific impacts on US jobs and salaries of outsourcing specific elements of health services to India [18,19], and the issue of redress in cases of malpractice [20]. Stack and Downing, [19] for example, develop a framework to examine what types of healthcare and IT jobs in the USA are most and least at risk to offshoring. Their framework integrates key variables - the changing need for proximity, the regulatory environment, firm-level security, intellectual property rights concerns, and emphasises the need to understand how the decisions to offshore are shaped by broad socio-political forces. They then use this to examine the routing of between 4 & 10% of the US medical transcription industry to India, the potential offshoring of Ask-a-Nurse programmes by managed care companies, and offshore outsourcing of professional interpretation of radiographic imagery. Kshetri [21] suggests that the micro and macro level impacts of offshore outsourcing in general are far from clear and uses institutional theory as a lens through which to consider the issues raised by existing literature, arguing that this allows us to understand the ‘macro level rules of the game’ For Kshetri outsourcing activities can be viewed as arenas of power relations in which various players engage in ‘institutional war’ and such an approach can illuminate the mechanisms by which regulative rules, social rules, culturally supported habits influence assessment, selection and continuation of outsourcing projects [21]*.*

### Transnational consumption of services

India is part of a growing Asian market for medical or health ‘tourism’, attracting consumers from developing and industrialised countries [16,22]. This topic has generated interest of late, twenty one papers published from 2004 to 2011 were found on this theme. The publications included medical and health related journals and social science journals, with a number of recent papers in *Global Social Policy*. Disciplinary perspectives include Management, Business Administration, Economics, Geography, Social Policy and Socio-legal studies. Overall the studies lack explicit theoretical orientation, and empirical studies were scarce until 2010. In all but one paper in this India-focused subset of a broader literature, medical tourism is considered from a health systems perspective. Connell [23] is the exception, taking a wider perspective from Geography to consider medical tourism as a niche market within tourism.

Several papers consider the implications of increasing medical tourism in India for national healthcare systems, either from the perspective of India or patients’ country of origin. The potential accentuation of problems and inequalities within the Indian healthcare system as a result of medical tourism is raised in several commentary and review papers [22,24,25]. Vijaya [26] draws on a small number of interviews with nurses to highlight how the medical tourism market may take away resources that otherwise would benefit the public health system in India. Some limited consideration is given to the implications of medical tourism to healthcare systems in the US and UK [27,28] and in these papers India is included as a major destination for those seeking care rather than the sole focus. In a further paper Cortez [29] provides a description of the legal system in India to assess what legal recourse is available to consumers if they fall victim to medical negligence when seeking care in another country.

Lunt and Carrera [30] provide a literature review on patient mobility from a European perspective but conclude that very little research is available in that area. However, our search does suggest that empirical social science research is increasing. Through thematic content analysis of marketing materials, Crooks et al. [31] study the messages and images used by companies to market India as a global health destination whilst Alsharif et al. [32] and Kangas [33] try to ascertain reasons for seeking care from the patients’ perspective. Overcoming challenges in identifying patients seeking cross-border care, Alsharif et al. [32] uses a questionnaire survey administered by health professionals in four countries. In contrast Kangas [33] builds detailed qualitative case studies to understand the drivers for patients from a low income country, Yemen, to seek care in India.

Twelve papers focus on India as a major destination for ‘reproductive tourism’ or travel. Ikemotot [34] argues that although reproductive tourism is a subset of medical tourism it is distinct in a number of important ways. Assisted reproductive technologies (ART) rely on women’s bodies or their reproductive labour, and if successful this results in the birth of a child, with implications that go beyond consumer’s interests alone [34]. We found a number of commentaries highlighting ethical issues arising from the different levels of regulation that make India a leading destination for ART [35,36]. Three papers from socio-legal studies [37,38] and social policy [39] perspectives consider how surrogate mothers in India will be protected through the currently proposed ART Bill. The tone of discussions across papers is generally cautionary and looking for greater restriction of practice, although Crozier [40] takes a rather different stance arguing that the cross-border providers of ova and surrogacy may be better protected by increasing the alternative options available to them rather than restricting their participation in this market.

A recent critical narrative review of literature highlights that most papers on cross-border reproductive care are based on commentary rather than empirical research [41]. Empirical research in the area of reproductive tourism is challenging but, as with medical tourism more broadly, there are recent studies attempting to overcome this. Surveys of fertility patients (in Canada, US and Australia) to identify the scope and volume of cross-border fertility services confirm that India is a major site of oocyte donation [42,43]. Pande provides the most substantial in-depth analysis with her study of surrogates in Gujarat [44,45]. Drawing on oral histories of 42 surrogates and participant observation she draws on feminist and global production theory to introduce the concept of ‘sexualised care work’ to describe commercial surrogacy, highlighting the parallels that can be drawn with other care work and with the stigma ascribed to sex work.

### Transnational movement of capital

Transnational movement of capital can take the form of loans or of foreign direct investment (FDI) for healthcare related provision. The very small literature in this area is dominated by perspectives from economics, and its size probably reflects difficulties in accessing data in this area. We identified six items published from 2001 onwards: two review articles, three articles and chapters using secondary data and two documents reporting on the findings from primary research. Together they shed light on the trends in this area over time. In the sole social policy piece Baru notes the role of the regional business groups and non-resident Indian doctors in promotion of the corporate sector in the southern cities of Hyderabad and Madras during the 1980s [46]. Smith indicates the evolution in the 1990s of new collaborations between Indian companies and multinational corporations such as Gleneagles based in Singapore, Royalton Medical Management based in Montreal, and Jardine Insurance from the UK [47]. In a subsequent paper Smith et al. report on the situation since 2000 in which India has allowed FDI in hospitals up to 100%, and they estimate that 90 FDI projects were approved (21 hospitals, 69 diagnostic centres) between 2000 and 2006, for a total US$53 million [16]. However, Chanda [48] argues that increased FDI inflows have been impeded by regulatory and structural impediments in the Indian economy.

With regard to external loans, Chakravarthi [49] highlights the role of the International Finance Corporation (IFC), a member of the World Bank Group, also in promoting private sector involvement in health care in India by providing loans to Indian corporates for their expansion plans and to set up hospitals in smaller cities and towns within the country. This expansion activity ranged from investment in existing facilities, joint ventures with local partners, or managed care services which integrate the financing and the delivery of medical services and alliances to develop healthcare networks and chains.

Lefebvre [50] and Chakravarthi [49] also describes how Indian FDI export has grown, citing as an example the Apollo Group of Hospitals which has franchises in several countries in Asia and Africa and partnerships with hospitals in the Gulf States, Mauritius, Tanzania, Nigeria, UK, Sri Lanka, Bhutan, Pakistan, and Bangladesh, as well as a telemedicine centre in Kazakhstan. Several authors highlight the difficulties of procuring data in this topic area and of discrepancies in definitions. Outreville’s international comparative study attempted to identify some of the determinants of foreign investment of the largest multinational companies (MNCs) and their relation with indicators of governance and stability, but encountered difficulty finding evidence on the relative importance of the largest MNCs operating in the healthcare sector [51]. Smith’s review article argues that this research area still requires greater clarity on what constitutes ‘FDI’ in order then to assess the costs and benefits and to match them against country objectives [47]. Alternative definitions might include proportion ownership, foreign or local management systems, and investment on an affiliate or non-affiliate basis. In addition, Smith suggests that it may be important to distinguish between ‘for profit’ and ‘not for profit’ FDI, between sub-sectors such as hospitals, insurance or other areas such as medical education, and, especially in context such as India, between involvement of non-resident nationals and ‘foreigners’ involvement in FDI.

Chanda’s work on the nature of foreign investment in hospitals in India stands out for its use of primary data [48,52]. Conducted for the World Health Organization, it utilises a survey of administrators and finance sections of 19 hospitals in 6 cities around the country, and semi-structured interviews with stakeholders ranging from health professionals to owners and managers of hospitals and nursing homes, health insurance companies, medical equipment suppliers and patients, civil society organizations and industry associations across three cities.

### Transnational movement of healthcare workforce

Migration has historically been the main pathway of health services trade, following colonial and linguistic ties, and these patterns remain but are complicated by stepwise migration [53] and by intraregional and circular migration [54]. Such areas have received some attention from the social sciences, particularly in the last few years. India has been, and remains, a major source country for migratory health professionals and we identified 29 papers, chapters and reports published from 1989 onwards that made reference to transnational movement of Indian health professionals and met our other selection criteria, with over half of these published in the period 2007–9. This was the largest volume of work of any of the themes and reflected a disciplinary range across Sociology, Geography, Management studies, Education, Economics and Social Policy.

The majority of articles that we indentified are commentaries and overview papers drawing together existing data to review emerging trends and the implication for workforce planning and development [54-62]. Chanda’s chapter in Blouin et al. [63] reviews a range of regional and bilateral labour, trade and economic cooperation agreements and also national recruitment codes, considering their objectives, scope and degree of legal compulsion. Dovlo’s overview paper [64] highlights the current interest in measuring and managing the migratory flow of health workers, in seeking reparations, payments or remittances, and in training substitute health workers. Kingma’s extensive work on nurse migration [54,60] brings together data from existing reports and studies to look at migration flows in the light of national nursing workforce imbalances, and to examine factors that encourage or inhibit nurse mobility, the potential benefits of circular migration, and nurse migration in the larger context of the global workforce. This synthesis is supplemented with interviews with nurse migrants on their motivations.

In order to understand migratory flows of physicians and nurses most authors draw on the data available from existing national professional governance systems and registers in receiving countries [16,65-68] and immigration systems [69]. Khadria’s description of the emergence of nurse recruitment hubs in India draws on ‘media reports’ and on data on nurses taking the US Commission on Graduate Foreign Nursing Schools examinations, to estimate the numbers of migrating nurses [70]. Only a few authors within our review had attempted comparative analyses for physician migration, using collated data from receiving countries [71] or a questionnaire to key informants in sending countries [65].

Nine of the twenty-nine papers and chapters report on empirical studies. These studies mainly focus on the motivations and experiences of the migrant doctor[69,71] or nurse [53-55,72-76]. A few papers deal with the cultural dimensions of migration of health workers. Robinson and Carey’s series of repeat in-depth interviews with Indian doctors working in the UK NHS explore the multi-leveled nature of explanations for international migration [69]. They suggest that while the movement of Indian doctors to the UK appears an economically driven phenomenon, migration also occurs because of “taken for granted” familial obligations rooted in the everyday cultural life and even the impressions generated by novels read in childhood. Three studies considered gender dimensions. Brush & Vasupuram [75] draw on existing literature to analyze the similarities and differences between skilled professional female migrants (nurses) and domestic workers (nannies and in-home caretakers) and how societal expectations, meanings, and values of care and ‘women’s work’, together with social, cultural, economic and political processes, construct the female migrant care-giver experience. Nair and Percot [53] draw on multisite ethnographic studies in Delhi and the Gulf States to elaborate the nuanced nature of stepwise migration for female Malayali nurses from Kerala. Finally, in her ‘global ethnography’ about the lives in the USA of migrant nurses from the state of Kerala in India, sociologist Sheba George uses the experience of these nurses to explore how contradictory cultural values alter the balance of gender relations between couples in the migratory process, when women who migrate before men become the breadwinners in the family, and how upheaval occurs not only in the families and immigrant community but also in the sending community in India [77].

Three papers problematise other conventional wisdoms around migration of health workers and take an overtly global framing for their analytical stance. Wright et al’s paper examines the historical antecedents to the current ethical debate around the ‘brain drain’ of health professionals [78]. S. Smith analyses the ‘microcosm responses’ adopted by one individual nation state, Australia, to the healthcare workforce shortage and argues for a coordinated global response with individual nation states being cognisant of the impact of their health policy, regulations and legislation on the ‘global community’ [79]. In one of the few papers to make explicit use of social science theory Raghuram, a geographer, offers an analysis of the dominant discourses around medical migration. In particular Raghuram focuses on the ways in which the brain drain discourse has privileged autonomous nation-states as the basis for, and the appropriate spatial unit for, thinking about the ethics of health care provision, and argues the case for a meso-level system whereby the benefits of skilled migration for destination countries would be calculated and an appropriate compensation paid to the countries from which migrants departed [80].

### Production, consumption and trade in specific healthcare related commodities

The search highlighted that the Indian healthcare economy is associated with a number of new developments in specific marketable niches of practice, therapy, technologies or research and development, we term these collectively as ‘healthcare related commodities’. Social science contributions to an understanding of this area could be found in relation to the organ trade [81-84] stem cell research and therapy [85-89], non-allopathic practices [90-93], biomedical clinical trials and biopharmaceutical research [94-100] and clinical by-products [101]. The review identified 21 articles in total published from 1998 to 2010. The main disciplinary perspectives include Political Science, Anthropology, Geography, Sociology, and History.

A number of editorials and overview papers chart India as a growing market for the different healthcare related commodities. In the case of organ trade and stem cell research this is noted to be facilitated by levels of legislation or weak enforcement of legislation [[82-85] and improvement to legislation is the focus of two editorials [81,98]. The implications of growth in trade within a globalized market are also considered. In the case of outsourcing clinical trials in India concerns are raised around safety and the public health interests of the Indian population [96,98] as well as the likely impact on development in outsourcing countries such as the US [94]. In the case of Ayurveda, concerns are raised that globalization and resulting standardisation have reduced Ayurveda from a system and overriding philosophy to itemised commodities to be traded on the global market [90,91], and Hoyez [93] using ethnographic research in India and France, analyses the importance of place to the practice of yoga through the concept of ‘therapeutic landscapes’.

A more in-depth understanding of globalization and the drivers and politics of these growing commodity markets, as well as potential implications for policy and for participants, is developed across the remaining papers. Salter & Salter [89] consider the political utility of bioethics in Human Embryonic Stem Cell (HESC) research. The paper analyses how the moral status of human embryos in particular creates the political need for bioethics to act as a vehicle for resolving cultural conflict and in further highlights the interests of countries such as India in adopting an approved bioethical position in order to legitimise its policy stance and global presences in HESC politics. With regard to stem cell research and regenerative medicine, Salter, a political scientist, demonstrates that in the absence of comprehensive resources countries such as India have to be selective about where they chose to intervene in order to maximise advantage. The explanatory concept of ‘speculative states’ is developed to understand India’s intervention in the regenerative medicine knowledge production process [87,88].

Patra & Sleeboom-Faulkner [86] examine how adult stem cell therapy is promoted in two leading centres in India. They use the concept of ‘bionetworking’ to examine how the recruitment of patients at local, national and global levels builds on the ‘political economy of hope’, from either patient or professional perspectives. In a similar vein, using a stakeholder analysis Muraleedharan [82] applies a politico-economic perspective to argue that the seemingly ineffective implementation of the Transplantation of Human Organs Act (1994) in India can be understood in the fact that all stakeholders can gain from the payment of organs and thus will continue to participate in the market. And in a highly original anthropological piece, Hodges [101] draws on cultural historical perspectives to understand the market in by-products of clinical encounters or ‘medical garbage’. The analysis shows how different ‘value chains’ of people arise and that risk has a crucial mediating role in the value of medical garbage and work undertaken to extract its value. A willingness to take on risk through the handling of waste is compensated for by the opportunity to earn money in direct economic exchange.

Finally, a body of sociological and anthropological work argues for a more critical understanding of participation in clinical trials [95,97,99,100]. For Prasad the rights and values of the population participating in trials should be understood in a transnational market in which India is a competitor [97]. Cooper [95] applies the concept of ‘experimental labour’ to account for some of the groundwork through which value is created in biomedical economies. Sunder Rajan focuses on ethics and argues that the harmonization of ethics goes with the harmonization of property regimes globally and as a result provide global capital that can turn “even healthy Indian populations into experimental subjects, who are both merely risked and free to choose to be so” 74: [100].

### New global governance issues and structures

Increased transnational activities have brought new transnational regulations and policies, some of which impact upon the healthcare arena in new ways. The search identified fourteen relevant articles, all published from 2000 onwards. The majority focused on the consequences for India of international agreements administered by the World Trade Organization (WTO) such as the agreement on Trade Related Aspects of Intellectual Property Rights (TRIPS) which sets minimum standards for intellectual property regulation and the General Agreement on Trade in Services (GATS). Disciplinary perspectives included Economics, Management, Law and Political Science. Sources ranged from *the Lancet* to social science journals and World Bank Reports.

One paper considered broader global health governance. From a socio-legal perspective, Gostin [102] provides a critical analysis of current global health architecture and need for change in order to meet basic health needs and to ensure greater country ownership and autonomy within health. With regards to TRIPS, common themes were the analysis of implications of new patent laws on access to medicines, particularly antiretrovirals [103,104] and of the emerging opportunities and challenges for pharmaceutical industries [105-110]. Papers consider how India has responded to TRIPS, how patent policies have been amended and implemented within India, and to what extent innovation and research and development has been stimulated following increased patent protection. There were two microeconomic analyses. Fink [107] calibrates a theoretical model with data from the pharmaceutical industry in India to simulate the possible impact of stronger patent policies on transnational corporations and in turn on market and welfare impacts, and Chadha [105] draws on product cycle and neo-technology theories of trade to analyse export performance of 131 Indian pharmaceutical companies.

Papers in this theme highlight the potential tensions between adherence to international agreements and achievement of national health goals, although India is given only limited focus within broader discussion. With regards to GATS, overviews by Chanda and Smith referred to earlier consider the implications of trade in health services, both positive and negative, for key health policy goals including equity, efficiency, quality and access [15,16]. Both point to the need for improved data collection and studies in order to answer issues raised by increased trade in health. Whittaker [36], in a case study of reproductive tourism in Asia, argues that GATS and current global regulation are not sufficient to address problems of exploitation and how to balance reproductive autonomy of individuals with the protection of rights of others.

Bloche [111] carefully analyses the laws and WTO’s response to argue that the potential threat to health and prioritisation of the protection of health within countries is misunderstood, and that there are social and political reasons why health protection has actually become more central to WTO even if not explicitly stated. In an exploratory discussion Chanda [52] is also more positive suggesting that GATS has further potential as a tool to ensure that the movement of health professionals is temporary rather than permanent and as a result be beneficial to host and source countries.

### Transnational diffusion of ideas and knowledge

The final theme concerns the transnational diffusion of ideas and knowledge, including policy ideas, political ideologies and discourses around health and healthcare in the context of India. In total there were nineteen articles (including three book chapters and two conference papers) and it is in this theme that India based scholars are particularly prominent. There was a strong emphasis on analysis of the influence of neoliberalism on health and healthcare delivery systems.

Published from 1998 to 2010, disciplines represented were primarily Social Policy, Sociology and Area and Development Studies, with smaller contributions from Management, Social Anthropology and Political Science. Journals of publication related to Health and Development, Gender Studies and Global Health, and Business Administration.

Two papers using contrasting quantitative and qualitative approaches attempt to study the impact of globalisation and economic reforms on the health of workers and reach similar conclusions that work and income are less reliable at the same time as out-of-pocket expenditure is increasing due to the growth in private health services [112,113]. Analysis from Social Policy shows that increasing privatisation of healthcare led to reductions in public expenditure on health and that this disproportionately impacts on vulnerable groups [46,113-116]. Rao [116] illustrates that whilst globalisation has led to international policy such as the Programme of Action promoting reproductive rights and health resulting from the International Conference on Population and Development (ICPD), diffusion of neoliberal ideas can at the same time erode the possibility of such gains, through the collapse of the India public health system. Two further papers consider the influence on neo-liberalism on autonomy and independence in decision making. Qadeer [117] argues that globalisation and uniformity of programmes means that it is very difficult for national governments in countries such as India to take autonomous decisions concerning reproductive health programmes, and Ramachandran [118] provides a critique of the reliance of NGOs on donors for project grants and how this has distanced them from their activist roots.

Two multi-country studies from political scientists examine the adoption of transnational policy ideas [119,120]. Through a systematic analysis of country scoping reports Holden [119] examines the export strategy of Public-Private Partnerships (PPP) from the UK. Shiffman [120] draws on qualitative case studies of five countries, including India, to understand what factors determine the political priority given to maternal mortality.

Donor and global discourses around HIV/AIDS and reproductive health forms another sub group within this theme and draw analysis from Sociology, Anthropology, Social Policy and Politics and International Studies. Sengupta [62] provides an analysis of the World Bank reproductive health programme in India, showing that the programme incorporates language used by feminist and radical groups who have opposed population control programmes in India but that the programme has not substantively changed. Finn & Sarangi [121] critically appraise the appropriateness of neoliberal, western-centric discourses on the attainment of health related quality of life and individual risk behaviours in the context of HIV in India. Using the UK’s Department of International Development and Norwegian’s Agency for Development Co-operation as case examples, Jones [122] tries to deconstruct donor policy to understand the predominate focus on prevention rather than treatment of HIV/AIDS. Misra’s ethnographic study examines how globally established scientific material is delivered locally through transnational exports and in turn how local translation feeds back to the global level [123].

The transnational reach of pharmaceutical companies is the focus of three studies within this theme. The first by Bower & Sulej focuses specifically on the growth of Indian pharmaceutical companies into western markets, examining the explanatory concepts of absorptive capacity, social capital and strategic alliances in cross-national contexts [124]. The second by Haakonsson uses global value chain analysis to understand the social and political pressures involved in production and trade to examine opportunities for upgrading pharmaceutical industry for which changes in Indian market provide important opportunities [125]. Ecks [126] introduces the concept of ‘near-liberalism’ to emphasise that elements of neo-liberalism, freedom, emancipation and autonomy are always held out as future goals. The anthropological case study of marketing of antidepressants shows how a company’s stated global corporate citizenship can be easily adapted in practice.

## Discussion

Using the lens of India, this literature review attempts to capture the ‘state of the art’ of social science literature concerning the evolving global healthcare landscape. Inevitably, there are several caveats. Such a far reaching topic as this does not lend itself to easy classifications. Despite the multiple search steps we cannot claim to have identified all the social science scholarship in this field, only that we have made a fairly comprehensive and systematic attempt. It is possible that some important work, most likely book chapters, will have eluded our methods of searching. The restriction to English language is an additional constraint. What became clear through our screening process is that the boundaries of what we determine to be ‘social science’ in the health sphere are porous. Ideas from the social sciences become mainstreamed (not least ‘globalisation’ itself). Social policy perspectives and social science research methods are taken up and employed by health professionals. We tried to make consistent decisions on where to draw our boundary for this review process, others might redraw it a little differently.

The specific focus on work related to a middle income country such as India both illuminates and restricts. A country focus did permit a more explicit search for work on the interface between the macro and the micro, the global and the local, than would have been feasible otherwise (our step 5). And it illuminates because it helps us to think through the inward and outward processes of transnational activity and their impact upon a specific locality. It restricts because it implicitly retains an orientation to the national state and because it may exclude seminal theory and empirical work that did not focus on that particular locality.

While it is impossible here to elaborate a research agenda for all the themes or all the social science disciplines here, several comments can usefully be made about what is still to be done and the research challenges of doing it. Firstly, the inadequacy of global data and the difficulties with definitions and data collection methods on all dimensions of health-related trade has been highlighted and this constraint was also apparent in much of the public health and health policy literature that we scanned and discarded during the selection process. This situation inhibits a proper understanding of global trends and comparative analysis, and requires attention by researchers and at a policy level. Secondly, although we found some evidence of cross-disciplinarity with reference made to other literatures from sister disciplines, the field is ripe for more cross-fertilisation. In particular we would endorse the call made by several key writers for empirical studies that integrate social and economic perspectives more explicitly. Third, the value of historical and contextualising analyses that unsettle the prevailing policy discourses has been demonstrated by a small number of scholars, but such work is scarce in spite of its clear relevance to India’s relationships with global forces.

Fourth, there is scope for many more empirical studies, and for greater testing and development of explanatory theory. In most thematic areas we found the majority of the literature to be overviews or commentaries. Quite a number of these did not move beyond the descriptive, although others did make careful and thorough critique of the available data. We did find interesting empirical work exploring the social processes inherent in the development and adoption of new technologies and those examine new transnational ways of working. It is perhaps the work grouped under the theme of ‘specific healthcare related commodities’ that offers some of the most illuminating social science work on health systems and globalisation within the body of literature that we have reviewed. Whilst the overview papers and commentaries point up potential ethical and governance problems, the body of anthropological and biopolitical research offers a rather deeper understanding of how actors at local, national and global levels can, for example, take advantage of unequal social and regulatory contexts to further stem cell research, and of the enactment of power and policy. Ethnographic studies of specific ‘products’ and of the production and reproduction of therapeutic landscapes are challenging and resource intensive but ultimately rewarding.

Underlying many of the preceding points is the broad question of how best social science researchers can connect the broad discussions of globalization with the local realities in health systems. Across the literature that we reviewed we saw very little comprehensive analysis of the kind that Holden and others have argued for elsewhere [127] – analyses that use multilevel data relating to the level of the firm or organisation, to the national level, to regional processes and institutions and to international organisations and agreements, and the interactions between these. Such an approach would seem to be a promising avenue for the future. The extended case study method [128], represented by only one study in this review [74], would also seem to have considerable potential for the study of globalisation and health topics. The ambition of the approach - as Burawoy puts it is ‘to extract the general from the unique, to move from the ‘micro’ to the ‘macro’, and to connect the present to the past in anticipation of the future’ 5:[128]. This may be what will be required to link large-scale processes, transnational actors, and the fine grain of everyday praxis.

This is a young and expanding field of study, with over half of the identified work published from 2007 onwards. Less than a fifth of the items included were reports of empirical studies. Transnational consumption of health services, the transnational healthcare workforce, trade in specific health-related commodities, and transnational diffusion of ideas have all received attention from social scientists in work related to India. Global health governance, transnational delivery of health services and the transnational movement of capital have received less attention. No one discipline or journal dominates the field - the diversity across specialist, multidisciplinary, social science and health journals is striking. *Social Science and Medicine* was the biggest single contributor, but with only six articles, followed closely by *Global Social Policy* and the Indian journal *The Economic and Political Weekly*. The body of work that we have reviewed on India illuminates many ways in which local healthcare systems are ever more interconnected and interdependent with each other, and how intricately they have become woven into the economic, political, social, cultural and technological dimensions of globalisation.

## Competing interests

The author(s) declare that they have no competing interests.

## Authors’ contribution

SFM and EP provided guidance on the framework and direction of the literature review. RB conducted the search, reviewed articles, prepared drafts of tables and drafted the methods section. EP reviewed articles contributed to the synthesis, wrote and redrafted manuscripts. SFM reviewed articles, contributed to the synthesis, and provided substantive inputs on drafts of the manuscript. All authors were involved in reading drafts of the manuscript and providing comments and suggestions for the paper. All authors read and approved the final manuscript.
